# Is pneumatic balloon dilation safe and effective primary modality of treatment for post-sleeve gastrectomy strictures? A retrospective study

**DOI:** 10.1186/s12893-018-0381-8

**Published:** 2018-08-02

**Authors:** Aneesh Shrihari Dhorepatil, Daniel Cottam, Amit Surve, Walter Medlin, Hinali Zaveri, Christina Richards, Austin Cottam

**Affiliations:** Bariatric Medicine Institute, 1046 East 100 South, Salt Lake City, UT 84102 USA

**Keywords:** Laparoscopic sleeve gastrectomy, Loop-duodenal switch, Strictures, Pneumatic balloon dilation, Endoscopic management, Bariatric

## Abstract

**Background:**

The optimal treatment of sleeve strictures has not been agreed upon at the current time. At our institution, we began using pneumatic balloon dilation to help resolve these obstructions in 2010. Herein we report our experience with pneumatic balloon dilation for the treatment of sleeve strictures.

**Methods:**

From Jan 2010 to Dec 2016 we retrospectively reviewed our prospectively kept database for patients who developed a Laparoscopic Sleeve Gastrectomy (LSG) stricture within 90 days of surgery. If the stricture was found, then we dilated all our patients initially at 30 mm at 10 PSI for 10-20 min (14.5 min average) and increased the balloon size (30-40 mm) and duration (10-30 min) in subsequent sessions if the first session was unsuccessful.

**Results:**

The review found that 1756 patients underwent either LSG or the first step of a Laparoscopic Duodenal Switch (LDS) (1409 LSG & 356 LDS). Of the 1756 patient 33 patients (24 underwent LSG, and 9 underwent LDS) developed a stricture as a complication of LSG. The average age of the patients was 46.4 (±9.6) years, and the average BMI was 43.7 (±6.4). The most common location for stricture was mid-body of the sleeve (54.5%). The average time from the primary surgery to diagnosis and first pneumatic dilation was 5.6 months (± 6.8) and 5.9 months (± 6.6) respectively. We successfully used pneumatic dilation in 31 (93.9%) of these patients to relieve the stricture.

**Conclusion:**

We conclude that pneumatic dilation is an effective procedure in patients with post sleeve gastrectomy stricture.

## Background

Prevalence of laparoscopic sleeve gastrectomy (LSG) for morbid obesity increased from 0 to 37% of the total world interventions for weight loss surgery between 2003 and 2013 [1]. This increase in popularity is attributed to its lower complication rates and safety as a procedure [2].

Despite the LSG’s rapidly rising popularity, strictures have remained an ongoing problem with an occurrence rate of 0.1–3.9% [1]. Strictures are usually divided into early strictures and late strictures. Patients with early strictures present within the first few weeks following the surgery complaining of dysphagia, vomiting, food intolerance, rapid weight loss, and regurgitation of either food or saliva. These are often pseudo strictures caused either as a result of post-operative edema or hematoma formation.

Late strictures, which occur > 1 month from the time of surgery are usually true strictures [3]. They are usually caused by ischemia, retraction due to scarring, or misalignment during stapling [3].

Treatment of LSG strictures is controversial. When to intervene, when to dilate, with what to dilate, and when to place metallic stents are all active areas of debate [4]. When these non-invasive methods fail, when to perform surgery and which type of surgery to perform, whether it’s a simple seromyotomy or strictureplasty or more invasive approaches to some bypass procedure, all lack consensus [5, 6].

In this article, we share one of the largest case series of LSG strictures treated with pneumatic balloon dilation as a primary modality of treatment. We also present a sustainable management plan using pneumatic balloon dilation as the primary modality of treatment for LSG strictures.

## Methods

This study has been approved by Quorum Review- Independent review board (QR# 31353), prior to data collection. 1409 cases of LSG and 356 cases of LDS were retrospectively reviewed from a prospectively collected database from Jan 2010 to Dec 2016. Demographic data for all patients was collected. This included pre-operative BMI, age, weight, and co-morbidities.

Thirty-three patients (1.8%) that presented with dysphagia, nausea, vomiting, or food intolerance after LSG or LDS with documented stricture on endoscopy or upper gastrointestinal (UGI) contrast study were considered eligible for pneumatic dilation. An area of narrowing, slow passage, or frank obstruction on an UGI were defined as a stricture. Similarly, a stricture was defined as a small, unsurpassable stretch of the lumen when using a 9.8 mm endoscope with or without symptoms, especially if the lumen is 6 mm or less [3]. We included all patients who demonstrated a stricture of gastric origin and underwent at least 20 mm of dilation into the study. We excluded all patients with strictures of non-gastric origin and patients undergoing a revision surgery of gastric bypass.

We used endoscopic pneumatic balloon dilation as a primary modality of treatment and repeated the procedure if symptoms did not resolve. Patients were informed of a surgical alternative in cases requiring repeated dilations.

### Technique of pneumatic dilation

We performed all our endoscopies under general anesthesia. Initially, an endoscope is passed down to the level of the stricture under direct vision. The stricture’s position is then confirmed on fluoroscopy and marked with a simple paper clip on the anterior abdominal wall. After confirming the stricture, the scope is then passed distally to the stricture and into the distal small bowel. A MAXXWIRE (MeritMedica Endotek) metallic guide-wire is passed through the scope and using fluoroscopic guidance placed in the distal small bowel, and the EGD scope is removed keeping the guide-wire in place. Then using the guidewire and fluoroscopic guidance we slide the Rigiflex II Balloon (Boston Scientific, Marlborough, Massachusetts) into position using the previously placed paper clip as a reference.

The balloon is then inflated under fluoroscopic and endoscopic visualization to 30 mm for an average of 14.5 min per patient. Optical view through the balloon and re-inspection endoscopy was used to confirm the resolution of the stricture. Depending on clinical improvement and the patients’ decision, repeat dilations were performed up to a maximum of 40 mm, with a minimum interval of 2 weeks between 2 dilations.

### Technique for LSG and LDS [7]

All surgeries were done by the three surgeons at the Bariatric Medicine Institute at a single hospital in Salt Lake City with identical technique. Out technique for both the LSG and LDS has been described previously [7].

Briefly, the LSG is created by stapling alongside a 40 French bougie. No patient in the study had his or her staple line oversewn or staple line reinforced. The staple line in all patients is started approximately 5 cm from the pylorus and ended at the angle of His. Each patient has a visual inspection of the hiatus to evaluate for a hiatal hernia with the simultaneous repair if a defect is found.

The loop duodenal switch procedure begins with an identical technique as a LSG. Following this, the gastroepiploic vessels are divided from the end of the sleeve staple line past the pylorus to the point where the perforating vessels from the pancreas enter the duodenum. This is 2 to 4 cm beyond the pylorus. The duodenum is divided with an Endo GIA stapler (Medtronic). Then the ileocecal valve is identified, and 300 cm of small bowel are measured and marked for point of anastomosis. The small bowel is then connected to the proximal duodenum.

## Results

Out of 1756 patients who underwent sleeve gastrectomy or LDS, 33 patients were included in this study. Twenty-seven patients were female, and 6 patients were male. The average age of the patients was 46.4 (±9.6) years, and the average BMI was 43.7 (±6.4).

Sixty-five percent of the patients followed up at 1 year and the excess body weight loss (EBWL%) at 1 year was 66.1% (62.6–69.7%).

24 patients underwent laparoscopic sleeve gastrectomy, and 9 patients underwent a LDS. Of 33 patients, 15 patients (45.4%), 5 patients (15.1%), and 9 patients (27.2%) had diabetes mellitus (DM), positive smoking history, and hiatal hernia repair, respectively. After endoscopy and/or Upper GI series, in 54.5%(18/33) patients the stricture was localized to the Mid-Body, 30.2%(10/33) were noted to have it at the incisura, and 15.2%(5/33) had it in the upper 1/3 of the sleeve. The average time from surgery to diagnosis was 5.6 months ±6.8 months. The average duration of primary surgery to the first pneumatic dilation was 5.9 months (+/− 6.6). The average duration from primary surgery to the second dilation was 8.6 months (+/− 7.1). Overall only 2 patients from the study group were dilated 3 times. The average duration of primary surgery to the third dilation was 19 months (+/− 19.7). The average balloon size used for the first dilation was 32.1 mm(+/− 6.8); for the second dilation it was 35 mm (+/− 5.2); and for the third dilation it was 40 mm.

The mean duration of dilation was 14.5 min (+/− 7.5 min). In the 21 patients who required a single dilation for symptom resolution, the mean duration was 15.6 min (+/− 6.6). Of the 8 patients that required a second dilation for symptom resolution, the mean duration of dilation in the first dilation was 14.6 min (+/− 8.3) and in their second dilation was 20 min (+/− 8 min). The 2 patients who required 3 dilations for symptom resolution were dilated for 10 min during the first dilation; 22.5 min (+/− 10.6 min) during their second dilation, and 30 min during their third dilation. 93.9% (31/33) patients had complete resolution of symptoms after pneumatic balloon dilation. 67.7% (21/31) required only 1 dilation, 25.8% (8/31) required 2 dilations, and 6.4% (2/31) required 3 dilations. Of the remaining 6.1% (2/33) patients, 1 patient required surgical intervention for symptom resolution, and 1 patient required a fcSEMS stent.

The location of the stricture, operative details, and the outcomes following pneumatic dilation can be seen in Table 1.

**Table 1:** Operative Details and Outcomes

Surgery		LSG			LDS			Total
N		24			9			33
MSS		5.1 ± 6.9			6.4 ± 6.3			5.9 ± 6.6
Operative Details of Dilations with avg. dilation (mm) and avg. duration (min)	1st Dilation	15/24 Dilation: 32 ± 7.7 Duration: 15.5 ± 6.3	7/24 Dilation: 31.4 ± 6.9 Duration: 15.2 ± 8.7	1/24 Dilation: 40 Duration: 10	6/9 Dilation: 31.6 ± 4.1 Duration: 15.8 ± 8	1 /9 Dilation: 30 Duration: 10	1/9 Dilation: 40 Duration: 10	
	2nd Dilation		7/24 Dilation: 33.7 ± 5.1 Duration: 20 ± 8.1	1/24 Dilation: 40 Duration: 30		1/9 Dilation: 30 Duration: 30	1/9 Dilation: 40 Duration: 15	
	3rd Dilation			1/24 Dilation: 40 Duration: 30			1/9 Dilation: 40 Duration: 30	
Resolution rate with each dilation	1st Dilation	62.5% (15/24)			66.7% (6/9)			
	2nd Dilation	29.1% (7/24)			11.1% (1/9)			
	3rd Dilation	4.1% (1/24)			11.1% (1/9)			
Overall Outcomes		Resolved: 95.8% (23/24) Unresolved: 4.1% (1/24)			Resolved: 88.9% (8/9) Unresolved: 11.1% (1/9)			Resolved: 93.9% (31/33) Unresolved: 6.1% (2/33)

*Abbreviations*: *N* number, *MSS* months since surgery, *LSG* Laparoscopic sleeve gastrectomy, *LDS* Loop duodenal switch

## Discussion

Strictures following a LSG is a very rare complication with a reported incidence of 0.1–3.9% [1]. Due to this rarity, there are few studies which thoroughly explore its management. Strictures usually present about 6 weeks after surgery [8], and are characterized by persistent dysphagia, nausea, vomiting, and food regurgitation following surgery. Early identification and management lead to better outcomes [8]. Our study is one of the largest case series detailing the magnitude of the complication and explores the efficacy of pneumatic balloon in its management.

According to many authors, the most common site of stricture is at the incisura [5, 9–11], but we observed that the most common location of the stricture was at the Mid-Body of the narrow sleeve (54.5%) followed by the incisura (30.2%) and then the upper 1/3rd of the sleeve (15.2%). This correlates with similar studies conducted by Donatelli et al. [12] and Rebibo et al. [13]. The question as to why we have more strictures in the mid-body just like Donatelli and Rebibo we believe relates to technique, specifically, the way the assistant pulls on the stomach while stapling. However, since the rate of stricture formation was only 2%, it will be several years and almost a thousand patients before we can definitively say whether or not our change in technique has made a difference.

Excess body weight loss achieved at 1 year after LSG can range from 50 to 70% [7, 14, 15]. We believe with early recognition and treatment, the presence of a stricture does not alter the pattern and degree of excess body weight loss achieved by LSG. This can be seen in the EBWL% achieved by the patients in this study, which was 66.1%.

Graded Pneumatic Balloon Dilation has been introduced as an effective and safer alternative to surgery for strictures [16, 17]. The primary aim of pneumatic balloon dilation is to pull apart the fibrosed muscular fibers. The pneumatic balloon, due to its rigid structure, achieves the high radial force of expansion [10]. Our study’s use of higher initial balloon size and increased duration time ensures the initial few minutes of dilation help tear the muscular fibers while the longer duration overcomes the elasticity of the fibrosis which has invariably occurred at the site of the stricture.

The magnitude of balloon dilation required for most effective management has not yet been defined. Most authors recommend 30 mm as the diameter of the balloon during the initial dilation for effective management of the stricture [3, 11]. Shnell et al. [10] demonstrated a relative lack of clinical success using the 20 mm balloon, and Donatelli et al. [12] and Rebibo et al. [13] showed higher success rates using the 30 mm balloon. In this study, we too used the 30 mm balloon during the first dilation. We believe dilating with up to 40 mm balloon is safe and effective even during the initial dilation. We dilated 8 patients with 40 mm balloons during their initial dilation and reported no adverse events associated with the pneumatic dilation. As far as efficacy is concerned, 50% (4/8) reported resolution of symptoms after a single dilation and 50% (4/8) required repeated dilation. In our experience, the 30 mm balloon is the safer and wiser choice as an initial dilator. We believe there isn’t a significant increase in efficacy by using a larger balloon as an initial dilator. The decision to use a larger balloon must be patient specific and should be taken after visualizing the stricture intra-operatively. Further studies comparing the 30 mm balloon and the 40 mm are required to determine the benefit of one over the other.

Compared to other studies, which document up to 1 min as the duration of dilation, this study has demonstrated a mean duration of 14.5 min (+/− 7.5 min). The increased duration of dilation leads to increased long-term patency of the previously strictured sleeve. We propose it leads to the lesser need for repeat dilations and lesser need for operative intervention to treat the stricture. We achieved successful resolution of symptoms in 93.9% of our patients only using pneumatic dilation. 67.7% of the patients with reported resolution of symptoms required just a single dilation. This could be the reason for our higher success rate as compared to other studies in the literature [9–12, 18]. Moreover, the patients who achieved symptom resolution after a single dilation were subjected to a longer duration of dilation (15.3 min) as compared to those who required a second dilation (14.6 min) {during their first dilation}. The longer balloon dilation times are not unheard of as Zundel dilated his sleeve strictures for a mean of 20 min [3]. In reality, the basic science and retrospective data are lacking in this regard, and there are no papers comparing dilation time and the incidence of complication. Our paper clearly showed that longer dilation times lead to better outcomes. However, we would never object to the argument that the appropriate dilation time and size are still open for vigorous debate.

Recently, Deslauriers et al. successfully performed dilation in 56% of his patients with sleeve stenosis [19]. Of these, 73% only needed single dilation, while 44% of the patients who were unsuccessful needed conversion to gastric bypass. All the successful cases needed a maximum of 3 interventions. Our success rate was higher and failure rate was lower than the one reported in this study. However, the success rate with single dilation was very similar between the two studies (73% vs 68%). Similarly, Nath A et al. [20] performed hydrostatic balloon dilation in 33 patients with gastric sleeve narrowing. He used 10–18 mm of balloon dilation for 1 min. Resolution was seen in 69% of his patients; 39.4% after first dilation, 15.2% after 2nd dilation and 15.2% after third dilation. Two patients (6%) had no improvement at all. His resolution rate was also lower compared to ours; however, it is important to note that size of his dilation was much smaller compared to the other published studies [10, 12, 13, 21].

Complex strictures sometimes require a different approach. The treatment options include fcSEMS stent and revision surgeries including pyloroplasty, stricturoplasty [22], or revision bariatric surgery [6]. fcSEMS are currently used for short periods to fine-tune narrowed sleeves [4]. Stents are most useful in refractory strictures, which have failed multiple attempts at dilation [4]. Unfortunately, stent migration is a very real complication and is reported in a very high number of cases [4]. This leads to repositioning in a majority of cases and operative stent removal in others.

In this study, we had 1 patient who required a stent. The patient underwent stent placement because of diverticulum formed due to gastric dilation, which required a partial gastrectomy. The patient experienced persistent pain and dysphagia 1 week after stent placement. After endoscopy, it was revealed that the stent had migrated into the esophagus, which had caused an intussusception of the esophagus and had led to an obstruction. We proceeded with endoscopic removal of the stent. The patient reported resolution of symptoms after removal of the stent.

Revision surgeries range from pyloroplasty, stricturoplasty, and seromyotomy to revision bariatric procedures like gastric bypass [6], single anastomosis gastric bypass, revision sleeve gastrectomy and loop duodenal switch. Dapri et al. [5] and Vilallonga et al. [11] have shown promising results of seromyotomies in patients suffering from long stenosis after sleeve gastrectomies. Some surgeons have approached the refractory strictures with more conservative techniques like a circular gastro-gastrostomy [23] or a laparoscopic median gastrostomy [24]. We had 1 patient who required revision surgeries for complete resolution of symptoms. The patient suffered from a refractory stricture at the incisura 1 month after a LDS procedure. After 3 unsuccessful attempts at graded pneumatic dilation over 6 months, a decision was taken to perform a partial gastrectomy to resect the gastric dilation caused by the stricture. This significantly controlled her symptoms. But one year later, the patient experienced a significant resurgence in her symptoms which led to the decision to perform a Heineke-Mickulicz stricturoplasty with a gastrogastrostomy. This procedure too had limited success. Finally, a decision was taken to place a transabdominal jejunostomy tube. Currently, the patient is still symptomatic but does not want a further intervention and is fine with her limited ability to eat as long as she has a functioning J tube.

In retrospect, our study could have been improved in many ways. These include the addition of a questionnaire to document resolution of symptoms like the BAROS (Bariatric Analysis and Reporting Outcome System) score [25] and a larger sample size.

Another major limitation of our study is the lack of long-term follow up for some of our patients. Though almost all patients at the time of writing do report resolution of symptoms, a follow up of at least 12 months is needed to rule out the possibility of recurrence.

Last but not least due to a small sample size, we were not able to thoroughly explore the management of refractory strictures. Larger studies are needed to compare fcSEMS versus revision surgery for the effective management of refractory strictures.

## Conclusions

The post-operative stricture is a rare complication following VSG, and earlier detection with effective management significantly reduces patient morbidity. Endoscopic treatment with pneumatic balloon dilation has repeatedly proven to be effective and safe as the first line of management for this complication.

In our series, the duration of dilation is as important as balloon size in achieving early resolution of symptoms and avoiding revision surgeries. However, both the timing and size of the balloon should not be considered settled, and we look for other authors to corroborate our findings or further define what can be done when strictures appear in the sleeve patient. Surgical intervention should be considered only after multiple failed attempts at dilation.

## Abbreviations

BAROS: Bariatric Analysis and Reporting Outcome System
DM: Diabetes mellitus
EBWL: Excess body weight loss
LAGB: Laparoscopic gastric band
LDS: Laparoscopic Duodenal Switch
LSG: Laparoscopic Sleeve Gastrectomy
UGI: Upper gastrointestinal series
